# Associations of Respiratory Distress Syndrome Severity and Other Factors With Transient Hypothyroxinemia of Prematurity

**DOI:** 10.7759/cureus.17159

**Published:** 2021-08-13

**Authors:** Mesut Dursun, Bahar Ozcabi

**Affiliations:** 1 Pediatrics and Neonatology, Biruni University Medical Faculty, Istanbul, TUR; 2 Pediatric Endocrinology, Memorial Bahçelievler Hospital, Istanbul, TUR

**Keywords:** prematurity, transient hypothyroxinemia, respiratory distress syndrome, multiple pregnancy, multiple surfactant use

## Abstract

Aim

This study examined the associations of respiratory distress syndrome (RDS) severity and other factors on thyroid hormone levels in very low birth weight (VLBW) infants.

Methods

The demographic characteristics, clinical course, morbidity, and initial thyroid function test results of VLBW infants diagnosed with RDS between July 2016 and September 2018 were obtained retrospectively. RDS severity was determined according to the requirement for multiple surfactants. Patients were divided into groups without and with hypothyroxinemia, and variables of interest were compared between the two groups.

Results

Our study involved 98 infants meeting the inclusion criteria; the incidence of hypothyroxinemia was 56.1%. Free T4 (fT4) levels were found to be negatively correlated with gestational week (p < 0.001) and birth weight (p < 0.001). The fT4 levels were significantly lower in infants requiring multiple surfactant doses. In infants with hypothyroxinemia, the duration of invasive mechanical ventilation and oxygen treatment was longer and hemodynamically significant patent ductus arteriosus, grade ≥ 3 intraventricular hemorrhage, and moderate to severe bronchopulmonary dysplasia were more common. Multiple pregnancy (odds ratio (OR) = 5.616, 95%; confidence interval (CI): 1.765-17.874) and the duration of invasive mechanical ventilation (OR = 1.05, 95%; CI: 1.005-1.096) were significant risk factors for the development of hypothyroxinemia in logistic regression analysis.

Conclusions

Transient hypothyroxinemia of prematurity is associated with RDS severity and early morbidities of prematurity. In the presence of multiple pregnancy, patients should be followed up more closely due to the possibility of hypothyroxinemia.

## Introduction

With developments in neonatology, mortality rates in premature infants are gradually decreasing. However, some morbidities are still of concern [1]. It is important that healthcare professionals dealing with premature infants ensure their optimal growth and neurodevelopment, rather than merely keeping them alive.

Thyroid hormones play a significant role in brain development [2]. The fetal hypothalamus-pituitary-thyroid axis starts to function after the first trimester, and its development is largely completed when term age is reached [3]. Thyroid hormone levels are lower in premature infants, especially those born before 30 gestational weeks (GW), for several reasons including the relatively low production and release of thyrotropic hormone, immaturity of the thyroid gland response to thyroid-stimulating hormone (TSH), insufficient iodine organification in thyroid follicle cells, and insufficient conversion of thyroxine (T4) into triiodothyronine (T3) [3].

Transient hypothyroxinemia of prematurity is characterized by low T4 and T3, and normal TSH levels, with thyroid function returning to normal during postnatal weeks six to eight [4]. Whether this condition, the incidence of which can reach 35-50%, plays a role in neurodevelopmental problems independent of other factors remains controversial [5,6].

Thyroid and various other hormones, together with glucocorticoids, affect lung development and surfactant production [7]. Moreover, respiratory distress syndrome (RDS) is significantly more common in very low birth weight (VLBW) infants treated for congenital hypothyroidism [8]. On the other hand, the presence of certain morbidities observed in premature infants (sepsis, shock, asphyxia, etc.) may impair thyroid hormone synthesis, which can result in lower hormone levels compared to healthy infants [3,8,9]. The effects of RDS, which is the leading cause of mortality and morbidity in VLBW infants, on thyroid function are not well known and the results of previous studies have been inconsistent [3,9].

We performed our study on infants with RDS at < 30 GWs in which the severity of RDS was determined according to the use of multiple surfactants, unlike previous studies. The aim of our study was to examine the effects of RDS severity and other possible factors on thyroid hormone levels.

## Materials and methods

This retrospective single-center cross-sectional study of premature infants hospitalized in the neonatal intensive care unit of Biruni University, Faculty of Medicine Hospital, between July 2016 and September 2018, was approved by the Biruni University Non-Interventional Research Ethics Committee before the study started (No. 2021/53-18).

Study group

The study group consisted of infants at GW < 30 with birth weight < 1,500 g who were hospitalized in the neonatal intensive care unit of our hospital. 

Inclusion criteria

Infants who showed clinical signs of RDS from birth, and therefore needed noninvasive or invasive mechanical ventilation for at least 24 hours, were enrolled in the study.

Exclusion criteria

The exclusion criteria were not needing invasive or noninvasive mechanical ventilation from birth, having a major congenital anomaly, being referred to another center without thyroid function tests (TFTs), being diagnosed with primary or secondary hypothyroidism, or death.

Data collection

The data of the infants included in the study, including GW, birth weight, mode of delivery, sex, antenatal steroid history, five-minute Apgar score, multiple pregnancy, small for gestational age (SGA), maternal age, maternal diseases (preeclampsia, gestational diabetes, chorioamnionitis, thyroid disease, etc.), duration of invasive and noninvasive mechanical ventilation, duration of oxygen treatment, surfactant requirement, use of drugs that suppress TSH such as dopamine or steroids, accompanying morbidities such as late-onset sepsis, grade ≥ 3 intraventricular hemorrhage (IVH), hemodynamically significant patent ductus arteriosus (HsPDA), stage ≥ 2 necrotizing enterocolitis (NEC), moderate to severe bronchopulmonary dysplasia (BPD), stage ≥ 3 retinopathy of prematurity (ROP), length of hospital stay, and mortality were extracted from patient files and online patient records. The initial free T4 (fT4) and TSH levels and fT4/TSH ratios were recorded based on venous blood samples, along with testing and treatment initiation times. 

Thyroid function tests

The fT4 and TSH levels were measured using an Architect i1000 analyzer (Abbott Laboratories, Abbott Park, IL, USA) employing the chemiluminescent microparticle immune study method.

The coefficient of variation (CV) for TSH was 1.9-5.2%, and the analytical sensitivity (AS) was 0.0025 µIU/mL; the CV for fT4 was 3.6-7.8% and the AS was 0.4 ng/dL.

The normal TSH level for infants born at GW 23-27 was considered as 3.5 ± 2.6 µIU/mL on postnatal day seven and 3.9 ± 2.7 µIU/mL on postnatal day 14; the normal fT4 level was considered as 1.47 ± 0.6 ng/dL on postnatal day seven and 1.45 ± 0.5 ng/dL on postnatal day 14 [2]. In infants born at GW 28-30, the normal TSH level range was considered as 3.6 ± 2.5 µIU/mL on postnatal day seven and 4.9 ± 11.2 µIU/mL on postnatal day 14; the normal fT4 level was considered as 1.82 ± 0.7 ng/dL on postnatal day seven and 1.65 ± 0.4 ng/dL on postnatal day 14 [2]. Subjects were divided into groups without hypothyroxinemia (group 1) and with hypothyroxinemia (group 2) according to their GW and fT4 levels on the day of examination. Demographic and clinical characteristics along with laboratory data were compared between the groups. To evaluate the association of RDS with TFT results, the patients were also classified into those requiring multiple doses of surfactant (> 1) and those not requiring multiple doses, with TFT results compared between the two groups. 

Respiratory management

Although the decisional process differs among physicians working in our unit, international guidelines were followed for target oxygen saturation, surfactant use, permissive hypercapnia, mechanical ventilation strategies and weaning approach, and postnatal steroid use [10]. High-frequency oscillatory ventilation (HFOV) was utilized only as rescue therapy. Minimally invasive surfactant application methods were not used in our unit during the study period. 

To minimize barotrauma and volutrauma caused by endotracheal intubation and mechanical ventilation, noninvasive ventilation strategies were preferred in infants with RDS, but pressure-controlled, volume-guaranteed synchronized conventional ventilation methods were used when invasive mechanical ventilation was required. 

During the study period, nasal continuous positive airway pressure (NCPAP) or nasal intermittent positive pressure ventilation (NIPPV) was used in infants during the post-extubation period. However, if NCPAP failed, NIPPV was applied before endotracheal intubation. All of the infants included in the study were given a loading dose of 20 mg/kg caffeine on the postnatal first day, followed by a maintenance dose of 5-10 mg/kg.

Definitions

Diagnosis of RDS was based on the presence of clinical signs of respiratory distress (two or more of the following: tachypnea, dyspnea, nasal breathing, intercostal retraction, oxygen requirement, and grunting), oxygen and/or positive pressure ventilation requirement, and radiological findings of RDS on chest X-ray (diffuse reticulogranular pattern, ground glass appearance, air bronchograms) [11].

The National Institutes of Health (NIH) consensus classification was employed for the diagnosis and classification of BPD [12]; Bell's criteria were used for the diagnosis of stage ≥ 2 NEC [13]; Papile's classification was employed for the diagnosis of grade ≥ 3 IVH [14]; the International Classification of Retinopathy of Prematurity was used for the diagnosis of stage ≥ 3 ROP [15]. Late-onset sepsis was diagnosed based on the presence of a positive blood culture after the third day of life [16].

Statistical analysis

Data were analyzed using the SPSS® software ver. 23.0 (IBM Corp., Armonk, NY, USA). The normality of the data distribution was examined using the Kolmogorov-Smirnov test. The chi-square test and Fisher's exact test were used to compare categorical variables between groups. The independent-sample t-test was used to compare normally distributed data between groups, and the Mann-Whitney U test was conducted to compare non-normally distributed data. The Pearson correlation coefficient was used to examine relationships between normally distributed quantitative variables, and Spearman's rho was employed to investigate relationships between non-normally distributed quantitative variables. Binary logistic regression analysis was conducted to identify risk factors for hypothyroxinemia. The results are presented as mean ± standard deviation or median (Q1-Q3) for quantitative data, and as frequency (percentage) for categorical data. The significance level was set at p < 0.05.

## Results

**Figure 1:**
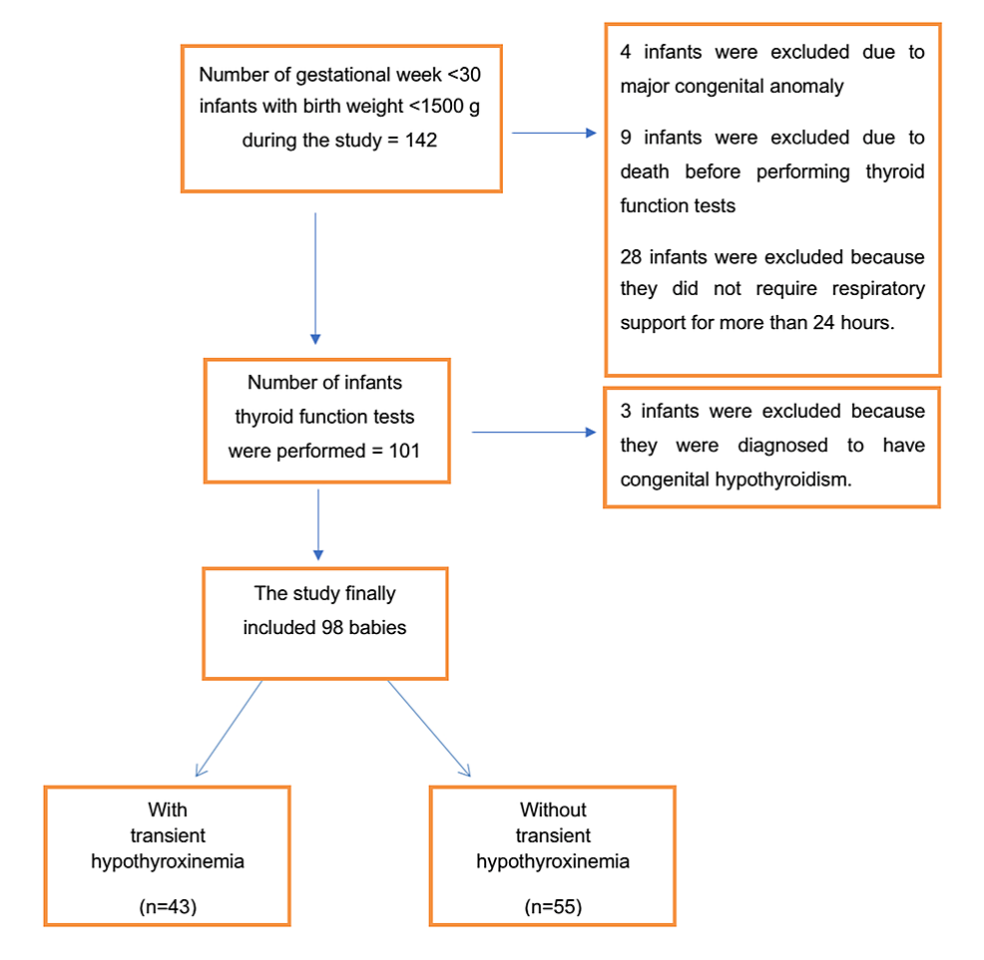
Flow diagram of participant enrollment

During the study period, 142 infants meeting the birth weight and GW criteria were hospitalized in our unit. The study enrolled 98 infants, of which 46 were girls, who met the inclusion criteria. Of these 98 infants, 56.1% (55/98) exhibited hypothyroxinemia. The flow chart for the study is presented in Figure 1. In group 1, the median GW was 27 (26-28), and the mean birth weight was 992.54 ± 227.29 g; in group 2, the values were 27 (25-28) and 952.27 ± 236.23 g, respectively. No differences were found between the groups in GW, birth weight, sex, mode of delivery, five-minute Apgar score, the presence of SGA, antenatal steroid use, preeclampsia, chorioamnionitis, or maternal characteristics (p > 0.05). However, the rate of multiple pregnancy was significantly higher among the infants in group 2 (p = 0.02). 

The clinical characteristics, neonatal morbidities, and TFT results of both groups are shown in Table 1. The rate of multiple surfactant use, duration of invasive mechanical ventilation, and duration of oxygen treatment were higher in group 2. Furthermore, HsPDA, grade ≥ 3 IVH, and moderate to severe BPD were more common, and the rate of postnatal steroid use was higher in group 2. No significant difference was detected between the groups in the timing of TFTs, TSH level, fT4/TSH ratio, or drugs used (p > 0.05). Because there was a significant difference between the groups in multiple pregnancy rates, infants born from multiple pregnancies and those born from single pregnancies were compared on all variables; no significant differences were detected (p > 0.05).

**Table 1:** Clinical characteristics and thyroid function test results HsPDA: hemodynamically significant patent ductus arteriosus; IVH: intraventricular hemorrhage; NEC: necrotizing enterocolitis; BPD: bronchopulmonary dysplasia; ROP: retinopathy of prematurity; MV: mechanical ventilation; TFT: thyroid function test; TSH: thyroid-stimulating hormone; fT4: free T4; fT4/TSH: free T4/thyroid-stimulating hormone ratio a Median (interquartile range); b Mean ± SD.

	Group 1 (n = 43)	Group 2 (n = 55)	p-value
≥ 2 surfactant doses, n (%)	9 (20.9)	24 (43.6)	0.018
HsPDA, n (%)	8 (18.6)	25 (45.5)	0.005
Grade ≥ 3 IVH, n (%)	5 (11.6)	16 (29.1)	0.037
Late-onset sepsis, n (%)	12 (27.9)	21 (38.2)	0.285
Stage ≥ 2 NEC, n (%)	3 (7)	7 (12.7)	0.505
Moderate to severe BPD, n (%)	8 (19)	23 (46)	0.006
Postnatal steroid, n (%)	16 (38.1)	35 (66)	0.007
Stage ≥ 3 ROP, n (%)	9 (21.4)	17 (32.1)	0.248
Duration of invasive MV^a^ (days)	3 (0–8)	6 (1–33)	0.004
Duration of noninvasive MV^a^ (days)	7 (3–18)	15 (4–22)	0.091
Duration of oxygen treatment^a^ (days)	9 (2–22)	22 (10–37)	0.005
TFT timing^ a^ (days)	11 (8–12)	11 (9–13)	0.476
TSH^a^ (mU/L)	3.59 (2.23–5.80)	3.13 (1.8–6.97)	0.574
fT4^b^ (ng/dl)	1.21 ± 0.21	0.78 ± 0.18	<0.001
fT4/TSH^a^	0.32 (0.2–0.6)	0.23 (0.12–0.49)	0.058
Length of stay (days)^a^	62 (53–77)	75 (55–107)	0.080
Death, n (%)	2 (4.7)	6 (10.9)	0.460

The fT4 levels decreased as the GW and birth weight decreased (r = 0.399, p < 0.001 and r = 0.364, p < 0.001, respectively). Also, the fT4 levels were significantly lower in infants requiring multiple surfactant doses. No group difference in the TSH level or fT4/TSH ratio was found (Table 2).

**Table 2:** TFT timing and TSH, fT4, and fT4/TSH ratio values, according to the use of multiple surfactants TFT: Thyroid function test; TSH: Thyroid-stimulating hormone; fT4: Free T4 a Median (interquartile range); b Mean ± SD.

	< 2 surfactant doses (n = 65)	≥ 2 surfactant doses (n = 33)	p-value
TFT timing^a^	11 (8–12)	11 (9–12)	0.901
TSH^a^	3.42 (1.95–6)	3.28 (2.17–7.3)	0.784
fT4^b^	1.06 ± 0.27	0.79 ± 0.24	0.000
fT4/TSH^a^	0.28 (0.18–0.6)	0.25 (0.13–0.44)	0.117

Multiple pregnancy (odds ratio (OR) = 5.616, 95%; confidence interval (CI): 1.765-17.874) and the duration of invasive mechanical ventilation (OR = 1.05, 95%; CI: 1.005-1.096) remained as significant risk factors for the development of hypothyroxinemia in multivariate logistic regression analysis, which also included GW, multiple surfactant use, multiple pregnancy, duration of invasive mechanical ventilation, grade ≥ 3 IVH, and HsPDA (Table 3).

**Table 3:** Risk factors for hypothyroxinemia revealed by binary logistic regression analysis MV: Mechanical ventilation; IVH: Intraventricular hemorrhage; HsPDA: Hemodynamically significant patent ductus arteriosus; OD: Odds ratio; CI: Confidence interval

	OR (95% CI)	p-value
Gestational age	1.301 (0.931–1.817)	0.123
≥ 2 surfactant doses	2.046 (0.689–6.075)	0.197
Multiple pregnancy	5.616 (1.765–17.874)	0.003
Duration of invasive MV	1.05 (1.005–1.096)	0.028
Grade ≥ 3 IVH	0.499 (0.097–2.572)	0.406
HsPDA	3.47 (0.927–12.994)	0.065

## Discussion

The present study demonstrated a relationship between transient hypothyroxinemia and some early morbidities of prematurity in VLBW infants. It was found that transient hypothyroxinemia was more common in infants with RDS who needed multiple surfactants. In addition, in the presence of HsPDA, grade >3 IVH, longer duration of invasive mechanical ventilation, and oxygen therapy, transient hypothyroxinemia was more common. Another remarkable result was that no significant difference was observed between the two groups in maternal or perinatal risk factors other than multiple pregnancy.

Transient hypothyroxinemia of prematurity is usually a self-limiting condition, with thyroid function returning to normal during postnatal weeks six to eight [3,4]. Different frequencies of hypothyroxinemia have been reported in the literature, and its incidence reportedly increases with decreased GW and the presence of accompanying morbidities [3,17]. The incidence of hypothyroxinemia in our study was higher than that reported in the literature, and no difference in GW or birth weight was found between infants with and without hypothyroxinemia. However, when we examined the correlations of GW and birth weight with fT4 levels, we found that fT4 levels decreased as the GW and birth weight decreased. We considered that the absence of group differences in GW and birth weight in our study was due to our use of different reference ranges for GW and postnatal age with respect to our definition of hypothyroxinemia. The high incidence of hypothyroxinemia may be because our study group comprised infants with RDS who needed invasive or noninvasive mechanical ventilation and were born at < 30 GWs. 

Various studies have demonstrated that non-thyroid diseases, such as respiratory distress, sepsis, IVH, HsPDA, and NEC, as well as some drugs, are associated with hypothyroxinemia of prematurity, and the degree of hypothyroxinemia is also correlated with the severity of such diseases [9,18,19]. However, the effect of RDS on thyroid function is not clear [3]. Williams et al. [9] found no significant relationship between RDS and hypothyroxinemia; they regarded this result as intuitive given that RDS is a time-limited, acute-stage disease associated with the use of antenatal steroids and postnatal surfactants. Carrascosa et al. [17] found no relationship between the frequency of RDS and hypothyroxinemia. However, it should be kept in mind that the present study was carried out on infants at GWs 30-36 with a relatively mild course of RDS. Moreover, many studies examining the relationship between RDS and hypothyroxinemia have not focused on the severity of RDS.

No definitive classification system is available for determining the severity of RDS, and studies performed to this end were generally conducted before the widespread use of surfactants [20]. In their retrospective study conducted on 8,024 infants born at GWs 22-28, Coshal et al. [21] found that infants using single or multiple doses of surfactant had higher rates of mortality and morbidities such as severe brain damage, BPD, and severe retinopathy than those who did not require a surfactant. They also reported that this relationship was dose-dependent and that the use of multiple surfactants could serve as a marker for respiratory immaturity and a means of identifying patients requiring careful follow-up to prevent ongoing lung damage and other adverse outcomes. A study carried out by Bilgin et al. on premature infants in Turkey [22] determined that the duration of mechanical ventilation, length of hospital stay, frequency of NEC, and mortality rates were higher in infants requiring multiple versus single surfactant doses. The present study assessed the severity of RDS according to the requirement for multiple surfactant doses. Although there was no difference in the TSH level or fT4/TSH ratio, fT4 levels were significantly lower in those requiring multiple versus single surfactant doses. 

Some studies have reported that multiple pregnancy increases the incidence of congenital hypothyroidism threefold and that it should be considered a risk factor in congenital hypothyroidism screening programs [23,24]. However, multiple pregnancy has not been definitively identified as one of the main risk factors for transient hypothyroxinemia. Therefore, our finding of a significant difference between groups related to multiple pregnancy was unexpected. Initially, we considered that this difference might reflect an increased risk of transient hypothyroxinemia among infants born from multiple pregnancies, but the results did not support this. Therefore, we concluded that preterm infants should be monitored more closely for transient hypothyroxinemia in cases of multiple pregnancy. 

Despite its life-saving capability, mechanical ventilation is associated with sepsis, ventilator-associated pneumonia, and BPD, and the risk of death and neurodevelopmental disorders increases with prolonged use [25]. In our study, the duration of invasive mechanical ventilation and oxygen treatment was longer in infants with hypothyroxinemia. Multiple surfactant use, HsPDA, grade ≥ 3 IVH, duration of invasive mechanical ventilation, GW, and multiple pregnancy (which can be characterized as early risk factors) were included in the multivariate logistic regression analysis. The duration of invasive mechanical ventilation and multiple pregnancy remained significant. Postnatal steroid treatment was not considered an early risk factor and was therefore not included in the logistic regression analysis; it is a late-term treatment used in some infants diagnosed with BPD. 

Limitations

The main limitation of our study was that it retrospectively examined patient records. Although international guidelines for the management of RDS were followed, the generalizability of the results of our single-center study is relatively low. Furthermore, there are no precise definitions of hypothyroxinemia of prematurity and no established criteria to evaluate the severity of RDS. 

## Conclusions

In conclusion, our study demonstrated that transient hypothyroxinemia was associated with some early morbidities of prematurity. Moreover, when the severity of RDS was determined according to the use of multiple surfactants, hypothyroxinemia was more common in infants requiring multiple surfactant doses. Finally, infants from multiple pregnancies should be followed up more closely given the possibility of hypothyroxinemia. Prospective randomized studies including more patients will provide additional valuable information about the risk factors for transient hypothyroxinemia of prematurity.
